# The health services burden of heart failure: an analysis using linked population health data-sets

**DOI:** 10.1186/1472-6963-12-103

**Published:** 2012-04-25

**Authors:** Jane Robertson, Patrick McElduff, Sallie-Anne Pearson, David A Henry, Kerry J Inder, John R Attia

**Affiliations:** 1 School of Medicine and Public Health, The University of Newcastle, Newcastle, Australia; 2 Hunter Medical Research Institute, The University of Newcastle, Newcastle, Australia; 3 UNSW Cancer Research Centre, University of New South Wales and Prince of Wales Clinical School, Sydney, Australia; 4 Institute for Clinical Evaluative Sciences and Department of Medicine, University of Toronto, Toronto, Canada; 5 Clinical Pharmacology, Calvary Mater Hospital, The University of Newcastle, Clinical Sciences Building, Waratah, NSW, 2298, Australia

**Keywords:** Heart failure, Hospitalization, Health services research, Australia

## Abstract

****Background**:**

The burden of patients with heart failure on health care systems is widely recognised, although there have been few attempts to quantify individual patterns of care and differences in health service utilisation related to age, socio-economic factors and the presence of co-morbidities. The aim of this study was to assess the typical profile, trajectory and resource use of a cohort of Australian patients with heart failure using linked population-based, patient-level data.

****Methods**:**

Using hospital separations (Admitted Patient Data Collection) with death registrations (Registry of Births, Deaths and Marriages) for the period 2000–2007 we estimated age- and gender-specific rates of index admissions and readmissions, risk factors for hospital readmission, mean length of stay (LOS), median survival and bed-days occupied by patients with heart failure in New South Wales, Australia.

****Results**:**

We identified 29,161 index admissions for heart failure. Admission rates increased with age, and were higher for males than females for all age groups. Age-standardised rates decreased over time (256.7 to 237.7/100,000 for males and 235.3 to 217.1/100,000 for females from 2002–3 to 2006–7; p = 0.0073 adjusted for gender). Readmission rates (any cause) were 27% and 73% at 28-days and one year respectively; readmission rates for heart failure were 11% and 32% respectively. All cause mortality was 10% and 28% at 28 days and one year. Increasing age was associated with more heart failure readmissions, longer LOS and shorter median survival. Increasing age, increasing Charlson comorbidity score and male gender were risk factors for hospital readmission. Cohort members occupied 954,888 hospital bed-days during the study period (any cause); 383,646 bed-days were attributed to heart failure admissions.

****Conclusions**:**

The rates of index admissions for heart failure decreased significantly in both males and females over the study period. However, the impact on acute care hospital beds was substantial, with heart failure patients occupying almost 200,000 bed-days per year in NSW over the five year study period. The strong age-related trends highlight the importance of stabilising elderly patients before discharge and community-based outreach programs to better manage heart failure and reduce readmissions.

## **Background**

Heart failure is associated with considerable morbidity and poor survival. It is characterised by numerous hospital readmissions and extensive use of health care resources [1-3]. The resulting substantial burden on health care systems and the associated costs are a consequence of ageing populations and improvements in the medical management of heart failure with the use of therapies such as beta-blockers, ACE inhibitors, aldosterone inhibitors and device therapies that prolong survival after ischaemic heart damage or heart failure related to hypertension and valvular heart disease.

There has been limited documentation of the health system impacts of heart failure in the Australian community. Morbidity estimates have typically been derived by applying international chronic heart failure incidence and prevalence data to Australian population estimates [4]. More direct estimates have been derived from the National Hospital Morbidity Data (NHMD) collection [5]. This information, however, is based on aggregated data collections, with no analysis at individual patient level.

Patient-level data are required to examine individual patterns of care, and differences in outcomes related to age, socio-demographic factors, and the presence of co-morbidities. In Australia, there is universal, publicly funded coverage for hospital and community-based medical services. Consequently, there are a number of administrative data sets that provide comprehensive coverage of patients with heart failure at the national or state level. Most experience with the analysis of linked health data-sets has been in Western Australia, which has validated the use of administrative data to identify patients with heart failure [6]. Although the rate of index hospital admission has fallen, the burden of disease has increased because of improved survival and the ageing of the community [7].

A capacity for linkage of publicly funded health administrative data sets at the level of the individual patient has recently become available in Australia’s most populous state, New South Wales (NSW). To inform planning for care of individuals in NSW suffering from heart failure we performed an analysis of the NSW linked data-sets. We measured the rates of index (first) admissions for heart failure, the readmission rates, mortality and median survival from first admission. To assess the burden of disease on the healthcare system, we examined the average length of stay (LOS) for the index admission, readmissions in the first year, and the impact of co-morbidities on readmission rates. Finally, we calculated the bed-days occupied by patients with heart failure during the study period.

## **Methods**

Access to the relevant NSW data-sets was through the Centre for Health Record Linkage (CHeReL; http://www.cherel.org.au).

### **Data sets**

Records were drawn from the NSW Admitted Patient Data Collection (APDC; representing all separations in public and private hospitals in NSW, including discharges, transfers and deaths) between 1 July 2000 and 30 June 2007; and death registrations (fact of death) in the NSW Registry of Births, Deaths and Marriages (RBDM) for the years 2000–2007.

### **Definitions**

#### *Heart failure cohort*

The cohort comprised all NSW residents aged ≥45 years with a first (index) admission for heart failure i.e. a principal hospital discharge (separation) code for heart failure (ICD-10 code I50) or hypertensive heart disease (I11, I13) in the APDC between 1 July 2002 and 30 June 2007. Hypertensive heart disease was included in the definition based on evidence that some cases of heart failure are classified under this rubric [8].

#### *Index admission for heart failure*

An index admission was defined by the absence of a heart failure separation code (any diagnostic position) in the two years prior to the eligible admission.

#### *Readmissions*

Readmissions were counts of patients re-presenting at NSW hospitals for any cause within one month (28 days) and one year after the index admission for heart failure. *Heart failure readmissions* were any readmission where the principal separation code was heart failure (I50) or hypertensive heart disease (I11, I13).

#### *Co-morbidities*

We used the validated Charlson Index as a co-morbidity measure using an algorithm based on the work of Sandarajaran [9]. Given that heart failure is included in the Charlson Index and all patients had heart failure, no point was given for this. Co-morbidities were assessed in two ways: firstly, by examining hospital separation codes (all positions) at the index admission; and secondly by including separation codes from the index admission and all admissions in the two years prior to the index admission combined.

#### *Burden of disease*

We calculated actual bed-days occupied due to index admissions and readmissions.

#### *Geography*

Geography was defined using the Accessibility/Remoteness Index of Australia (ARIA) [10].

#### *Socio-economic status*

Socio-economic status was defined using the SEIFA (Socio-Economic Indexes for Areas) Index [11] based on 2006 Australian Census responses, and divided into quintiles.

### **Data linkage**

We used extracts of linked hospital separation data from the APDC with RBDM death registrations with encryption to protect the identity of individual patients. Data linkage by the CHeReL uses probabilistic matching of patients’ names and other identifiers with *ChoiceMaker* software [12], supplemented with clerical review of doubtful matches.

### **Statistical methods**

Age-specific rates of index (first) admissions for heart failure or hypertensive heart disease to public and private hospitals in NSW (1 July 2002–30 June 2007) were calculated by dividing the number of index events in 5-year age groups by the NSW population in that age group in that year. Age-standardised rates and 95% confidence intervals were calculated for each year using the indirect method with the 2006 NSW census population as the reference. Trends over time were examined with linear regression models.

The proportions of patients with a readmission (any cause or heart failure) in the first month (28 days) and first year post-index admission were calculated for the whole population and by 5-year age groups. The probabilities of readmission at one-month and one-year were estimated using Kaplan-Meier curves and the log rank test was used to test for statistically significant differences between age groups; individuals were censored at the time they died.

The LOS for the index admission and re-admissions was summarised as the mean number of days (with standard deviation), and presented for the whole population and by 5-year age groups. Linear regression was used to examine differences in LOS between age groups, after adjusting for potential confounding variables (age, gender, Charlson Index based on hospital separation codes for the index admission, geography using the ARIA index, and socioeconomic status using SEIFA quintiles). LOS data are not normally distributed (highly skewed to the right) and a robust variance estimator was used to correct for this. Cox proportional hazards modelling were used to assess the impact of potential risk factors for readmission to hospital. Where there was a missing value for any outcome or co-variate, the record was excluded from the analysis; this affected fewer than 3% of observations in the dataset. We tested the assumption of proportional hazards by examining the graph of the log(−log(survival)) versus log survival time graph.

Mortality rates and median survival time were calculated from first admission for heart failure or hypertensive heart disease. Survival from index admission to death was estimated using Kaplan-Meier curves, with curves fitted by 5-year age groups. The log rank test was used to test for statistically significant differences between age groups.

All analyses were done using SAS version V9.1 (SAS Institute Inc., Cary, NC, USA) and analyses performed at the 5% significance level.

The study was approved by the NSW Population and Health Service and University of Newcastle Research Ethics Committees.

## **Results**

There were 14,972,359 hospital separations in the APDC between 1 July 2000 and 30 June 2007, 67,018 of which had a principal diagnosis ICD-10 code of I50, I11 or I13, representing 41,904 persons. Of these, 29,735 persons had not been hospitalised with these separation codes (any position) in the two years prior to this event. Removing the 574 people aged <45 years at the index admission left 29,161 persons (with 645,245 separations) to form the heart failure study cohort. Patients with hypertensive heart disease comprised <2% of the study population.

### **Demographic characteristics**

Most patients (67.9%) were ≥75 years of age and 28.7% were aged ≥85 years (Table 1). There were approximately equal numbers of males and females in the cohort (14,604 males, 14,557 females). Females represented around one-third of those aged 45–64 years, but just under two-thirds (63.5%) of those aged ≥85 years (data not shown).

**Table 1:** Number and percent of index admissions by gender, age group, marital status, diagnosis and ARIA and SEIFA indices

	2002 – 03*	2002 – 03*	**2003-04**		**2004-05**		**2005-06**		**2005-06**	**2006-07**
	**N**^**†**^ **= 5854**		**N = 5935**		**N = 5606**		**N = 5813**		**N = 5953**	
	**n**	**%**	**n**	**%**	**n**	**%**	**n**	**%**	**n**	**%**
**Sex**										
Male	2899	50	2924	49	2804	50	2983	51	2994	50
Female	2955	50	3011	51	2802	50	2830	49	2959	50
**Age Group**										
45-49	68	1.2	61	1.0	54	1.0	88	1.5	81	1.4
50-54	114	1.9	136	2.3	105	1.9	127	2.2	122	2.0
55-59	206	3.5	201	3.4	191	3.4	199	3.4	212	3.6
60-64	302	5.2	349	5.9	302	5.4	317	5.5	324	5.4
65-69	472	8.1	463	7.8	478	8.5	423	7.3	408	6.9
70-74	800	14	760	13	683	12	643	11	662	11
75-79	1085	19	1082	18	1019	18	1054	18	1000	17
80-84	1256	21	1205	20	1209	22	1205	21	1316	22
85+	1551	26	1678	28	1565	28	1757	30	1828	31
**Marital Status**										
Married (including defacto)	2686	46	2755	46	2705	48	2764	48	2797	47
Never married, widowed, divorced, separated	2951	50	2932	49	2692	48	2872	49	3017	51
Unknown	214	3.7	241	4.1	180	3.2	176	3.0	138	2.3
**Diagnosis**										
Hypertensive heart disease (I11, I13)	106	1.8	84	1.4	86	1.5	84	1.4	83	1.4
Congestive heart failure (I50.0)	3918	67	4018	68	3848	69	4151	71	4365	73
Left ventricular failure (I50.1)	1614	28	1633	27	1496	27	1389	24	1283	22
Heart failure, unspecified (I50.9)	216	3.7	200	3.4	176	3.1	189	3.3	222	3.7
**ARIA**‡										
Highly Accessible	4264	75	4393	76	4164	76	4315	76	4394	76
Accessible	1159	20	1131	20	1022	19	1081	19	1099	19
Moderately Accessible	206	3.6	194	3.4	217	4.0	186	3.3	178	3.1
Remote / Very Remote	72	1.3	65	1.1	52	1.0	72	1.3	76	1.3
**SEIFA**‡										
Most Disadvantaged (1)	784	14	771	13	666	12	759	13	713	12
2	985	17	1005	17	942	17	965	17	1042	18
3	1308	22	1356	23	1292	23	1326	23	1313	22
4	1220	21	1243	21	1242	22	1264	22	1299	22
Most Advantaged (5)	1526	26	1528	26	1438	26	1452	25	1514	26

* Financial Year (1 July – 30 June).

† N = number of persons with index admissions.

‡ Data not available for all cohort members.

### **Comorbidity burden**

Patients had a median of 2.0 comorbidities recorded at baseline admission, although the range was wide (0–13, not including heart failure), with some evidence of an increase in comorbidity burden over time (Table 2). Re-calculation of the Charlson Index from hospital separation codes at the index admission and all admissions in the previous two years combined did not change the estimates substantially. Across the cohort this had the effect of increasing the mean number of comorbidities per patient by 0.5, with the median number of recorded comorbidities increasing from 2.0 to 3.0.

**Table 2:** Co-morbidity burden assessed by Charlson Index

Variable	**Statistic**	2002 – 03*	**2003 - 04**	**2004 - 05**	**2005 -06**	**2006 - 07**
		**(N = 5854)**	**(N = 5935)**	**(N = 5606)**	**(N = 5813)**	**(N = 5953)**
Charlson Score	mean (sd)	2.2 (1.5)	2.2 (1.5)	2.5 (1.6)	2.3 (1.5)	2.4 (1.6)
(based on index admission)	median	2.0	2.0	2.0	2.0	2.0
(q1, q3)	(q1, q3)	(1.0, 3.0)	(1.0, 3.0)	(1.0, 3.0)	(1.0, 3.0)	(1.0, 3.0)
Charlson Score	mean (sd)	2.7 (1.8)	2.8 (1.9)	3.0 (2.0)	2.8 (1.9)	2.9 (2.0)
(based on two years history)	median	2.0	2.0	3.0	3.0	3.0
(q1, q3)	(q1, q3)	(1.0, 4.0)	(1.0, 4.0)	(1.0, 4.0)	(1.0, 4.0)	(1.0, 4.0)

* Financial Year (1 July – 30 June).

† N = number of persons with index admissions.

sd = standard deviation; q1,q3 = quartile 1, quartile 3.

The most commonly recorded co-morbidities at the index admission were diabetes with chronic complications (27% of patients), pulmonary disease (15.6%), renal disease (14.2%), dementia (5.9%), acute myocardial infarction (5.3%), peripheral vascular disease (4.2%), and cerebral vascular disease (3.4%).

### **Age-specific and gender-specific rates of index admissions for heart failure**

The rates of an index admission for heart failure increased consistently with increasing age; and were higher for males than females for all age groups (Table 3). Index admission rates decreased over time; the age-standardised rates for males decreased from 256.7 to 237.7 per 100,000 from 2002–3 to 2006–7 and for women from 235.3 to 217.1/100,000. These trends over time were statistically significant (p =0.0073, adjusted for gender).

**Table 3:** Age-specific and age-standardised rates of index admission by gender and financial year

		**Rate of admission (per 100000 persons aged ≥45 years)**				
**5 Year Age group**	**Gender**	2002 – 03*	**2003 – 04**	**2004 – 05**	**2005 – 06**	**2006 – 07**
45-49	Male	19.4	16.2	14.7	27.8	20.9
n = 352	Female	9.9	9.7	7.9	8.6	12.1
50-54	Male	33.8	37.0	30.8	40.6	39.1
n = 604	Female	19.1	25.9	17.4	17.2	15.5
55-59	Male	70.8	65.4	64.5	63.1	67.2
n = 1009	Female	38.2	37.3	30.7	33.8	34.6
60-64	Male	132.8	155.7	131.5	137.3	130.8
n = 1594	Female	75.8	79.3	64.5	60.6	61.9
65-69	Male	236.0	226.2	237.9	211.5	190.1
n = 2244	Female	152.1	147.5	139.9	116.1	119.0
70-74	Male	413.8	432.9	391.6	357.5	345.8
n = 3548	Female	311.9	271.6	249.7	247.1	266.8
75-79	Male	691.5	678.1	620.0	686.9	652.6
n = 5240	Female	493.7	486.3	466.2	442.4	420.4
80-84	Male	1165.3	1092.9	1026.5	1058.1	1133.1
n = 6191	Female	886.5	799.5	792.6	729.2	774.5
85+	Male	1950.3	1864.6	1781.1	1871.3	1784.9
n = 8379	Female	1430.5	1602.6	1393.9	1493.9	1496.0
Age Standardised	Male	256.7	252.6	236.1	244.1	237.7
		(247.4, 266.1)	(243.4, 261.7)	(227.4, 244.8)	(235.4, 252.8)	(229.2, 246.2)
Rate	Female	235.3	236.8	215.7	213.2	217.1
(95% CI)		(226.8, 243.8)	(228.3, 245.2)	(207.8, 223.7)	(205.4, 221.1)	(209.3, 224.9)

* Financial Year (1 July – 30 June).

### **Hospital readmissions and deaths**

**Figure 1:**
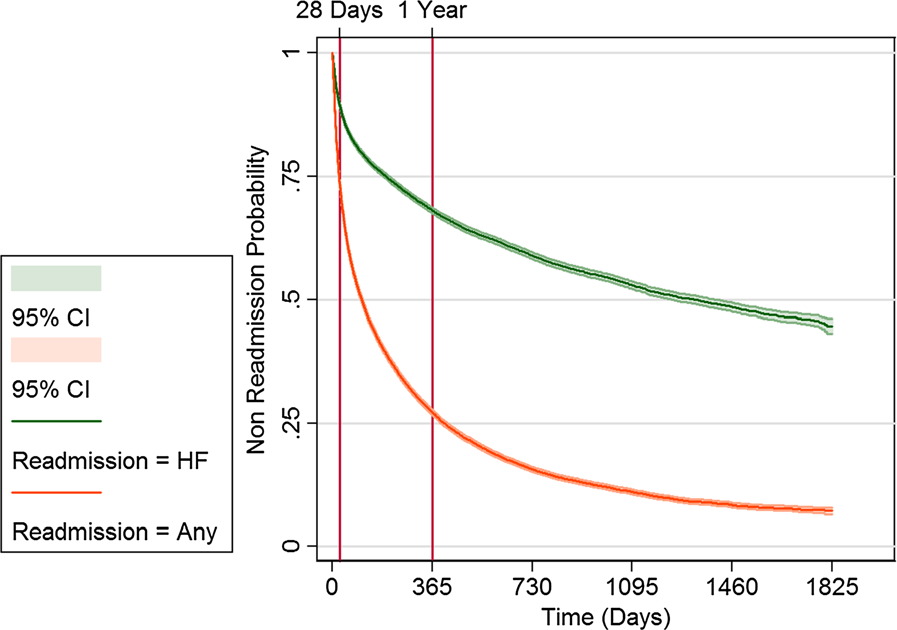
Kaplan-Meier curves for time to heart failure readmission and time to any readmission.

Of the 29,161 patients, 7,415 had a readmission (for any cause) within 28 days; 18,493 had one or more readmissions within one year of the index admission, giving readmission rates of 27% and 73% at 28 days and one year respectively (Table 4). Readmissions attributed to heart failure were 11% and 32% at 28 days and one year respectively (Figure 1). The proportions of readmissions attributed to an exacerbation of heart failure increased with age (Table 5). Heart failure readmission rates at one year increased by age from approximately 25% in the 45–49 year age group to 37% in people aged ≥85 years (data not shown).

**Table 4:** Patients with specified events and risk for events within 28 days and 1 year of the index admission

**Outcome**	**At 28 days**		**Within 1 year**	
**Number of Persons**	**Number of Persons**	**Probability of Outcome**^**†**^	**Number of Persons**	**Probability of outcome**^**+**^
Re-admission for any cause	7415	0.27	18493	0.73
Readmission - heart failure*	3007	0.11	7848	0.32
All-cause mortality	2531	0.10	6890	0.28
Readmission or death	9471	0.35	21125	0.79
Readmission HF or death	5302	0.20	12556	0.49

* heart failure or hypertensive heart disease as principal separation code.

^**†**^ derived from Kaplan-Meier curves.

**Table 5:** Proportion of readmissions attributed to heart failure within 28 days and one year of the index admission

**Age Group**	**Readmissions within 28 days**			**Readmissions within 1 year**		
	**Any cause**	Heart Failure*	**% of readmissions**	**Any cause**	Heart Failure*	**% of readmissions**
Whole population	7415	3007	41	18493	7848	40
45 – 49	107	36	34	221	80	36
50 – 54	155	48	31	366	124	34
55 – 59	288	76	36	663	219	33
60 – 64	423	131	31	1012	378	37
65 – 69	598	210	35	1447	575	40
70 – 74	905	321	36	2274	911	40
75 – 79	1297	496	38	3453	1425	41
80 – 84	1565	656	42	3985	1717	43
85+	2077	1033	50	5072	2419	48

* heart failure or hypertensive heart disease as principal separation code.

**Figure 2:**
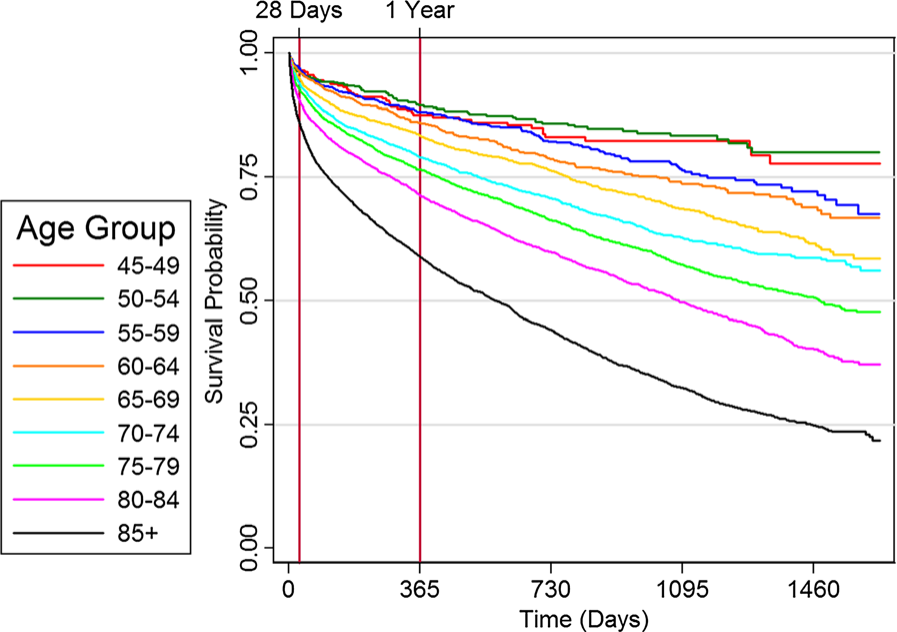
Kaplan-Meier curves for time to death for each 5 year age group for patients over 45 year of age.

All-cause mortality rates were 10% at 28 days and 28% at one year (Table 4). As expected, there were age-related trends, with 28 day and one year mortality increasing from 4.1% and 13.1% in those aged 45–49, up to 14.9% and 41.8% in those aged ≥85 years. Median survival after a heart failure index admission ranged from about 1.5 years in those aged over 85, three years for those aged 80–85, 4 years for those aged 75–79 to even longer for those younger than 75 (Figure 2).

### **Length of stay**

There were strong and statistically significant age-related trends for LOS for the index admission, readmissions for any cause and heart failure readmissions (Table 6). Readmissions for heart failure were longer than readmissions for any cause for the entire cohort (8.3 versus 4.8 days) and for all age groups. In the case of those aged ≥85 years, the mean duration of a heart failure readmission was comparable to that of the index admission (9 versus 9.6 days).

**Table 6:** Mean length of stay in days for index admission and readmissions

**Age group**	**Number of patients**	**Mean LOS in days**		
		**Index admission Mean (SD)**	**Readmission for any cause Mean (SD)**	Heart failure readmission* Mean (SD)
Whole population	29161	7.8 (18.1)	4.8 (12.1)	8.3 (12.1)
45 – 49 years	352	5.9 (6.5)	2.1 (6.0)	5.7 (7.6)
50 – 54	604	6.3 (7.9)	2.7 (5.5)	7.0 (7.2)
55 – 59	1009	5.9 (6.7)	2.4 (6.1)	8.0 (12.1)
60 – 64	1594	6.2 (6.0)	3.3 (7.5)	7.4 (8.3)
65 – 69	2244	6.6 (7.2)	3.6 (8.1)	7.9 (9.4)
70 – 74	3548	6.9 (7.8)	3.9 (9.0)	8.1 (9.8)
75 – 79	5240	7.2 (7.3)	4.7 (11.5)	8.2 (12.3)
80 - 84	6191	7.6 (9.5)	6.0 (12.8)	8.4 (9.5)
85+	8379	9.6 (31.2)	8.5 (20.4)	9.0 (15.8)

* heart failure or hypertensive heart disease as principal separation code.

### **Burden on hospital health services**

There were a total of 954,888 hospital bed-days for any cause over the 5 years of this study (Table 7). Of these, 383,646 hospital bed-days were specifically for heart failure, with 321,281 (83.7%) of these occurring in the first year post index admission. Of those in the first year, 106,679 (33.2%) of the bed-days related to subjects aged ≥85 years.

**Table 7:** Hospital bed-days occupied during study period (2002–2007)

		**Number of hospital bed-days**	**Number of hospital bed-days**	
**Age group**	**Gender**	Number of subjects* N = 29161	**Index admissions + readmissions any cause**	**Index admissions + readmissions for heart failure**
45-49	Male	236	7 799	2 121
Female		116	3 803	1 117
50-54	Male	396	9 484	3 739
Female		208	5 587	2 133
55-59	Male	664	24 613	7 489
Female		345	12 247	3 852
60-64	Male	1 068	36 294	12 262
Female		526	17 004	5 737
65-69	Male	1 374	48 279	17 634
Female		870	30 220	10 182
70-74	Male	2 015	65 630	24 480
Female		1 533	55 206	19 534
75-79	Male	2 830	96 495	36 486
Female		2 410	84 129	31 738
80-84	Male	2 971	98 375	39 050
Female		3 220	108 054	44 089
85+	Male	3 050	95 002	46 032
Female		5 329	156 667	75 971
Total	Male	14 604	481 971	189 293
Female		14 557	472 917	194 353

### **Risk factors for hospital readmission**

A Cox regression model indicated that the major risk factors for any readmission were increasing age, increasing Charlson score, and male gender (Table 8).

**Table 8:** Cox regression models with time to any readmission as the outcome

**Crude**	**Crude**		Adjusted*		
**Hazard Ratio**	**Hazard Ratio**	**95% CI**	**Hazard Ratio**	**95% CI**	**p†**
**Sex**										
Male	1		1							
Female	0.95	0.92, 0.98	0.93	0.89, 0.96	<.0001					
**Age Group**										
< 55	1	1								
55-59	1.10	0.99, 1.22	1.03	0.90, 1.18	0.6189					
60-64	1.07	0.98, 1.18	1.00	0.89, 1.13	0.9971					
65-69	1.12	1.03, 1.23	1.03	0.92, 1.16	0.5847					
70-74	1.12	1.03, 1.22	1.05	0.94, 1.17	0.3873					
75-79	1.21	1.12, 1.31	1.12	1.00, 1.24	0.0437					
80-84	1.22	1.12, 1.32	1.15	1.03, 1.28	0.0101					
85+	1.23	1.13, 1.33	1.14	1.02, 1.27	0.0167					
**Charlson Index**	1.06	1.06, 1.07	1.07	1.06, 1.09	<0.0001					
**Index Length of stay**	1.00	1.00, 1.00	1	1.00, 1.00	0.0006					
**Hypertension**										
No	1		1							
Yes	1	0.97, 1.02	0.98	0.95, 1.02	0.4133					
**Depression**										
No	1									
Yes	1.01	0.91, 1.12	0.99	0.85, 1.14	0.8582					

* models were adjusted for age, sex, marital status, ARIA category, SEIFA quintile, financial class, hospital type, Charlson Index, length of stay, hypertension, depression.

† P-values and confidence intervals calculated in SAS.

## **Discussion**

This study used linked administrative data to provide a picture of the typical trajectory of a heart failure patient from the time of initial admission, and to demonstrate the health system impacts of heart failure in New South Wales. The most important observations were the high rates of hospital admission to hospital, the age dependence of incidence rates and the 7-8% reduction in the rates of index admissions noted over the study period. There is substantial morbidity after a diagnosis of heart failure, with consequent health system impacts due to high readmission rates for heart failure, and long durations of hospital stay, particularly in the older age groups. Mortality was high at one year, although many deaths were not attributed to heart failure.

Consistent with other studies [2,5,13], we demonstrated a more than 10 fold increase in index admission rates for those aged ≥65 years compared to those aged 45–64 years. Incidence rates were higher for males than females at all ages, though the longer female life expectancy meant there were more females than males in the cohort in the oldest age groups (≥ 80 years). Age-standardised rates decreased over time in both males and females, a finding consistent with other studies and settings [7,14-16].

Direct comparisons of our estimates with other studies are challenging because of differences in the data sources used and the standardisation applied. Based on 4812 index admissions for heart failure between 2002 and 2005 in Western Australia, Teng et al [7] concluded age-standardised admission rates for heart failure of 249/100,000 and 176/100,000 for men and women respectively, with 41% of admissions in those aged <75 years. We found only 34% of index heart failure admissions were in those aged <75 years, and our age standardised rates were higher at 257 and 235 per 100,000 for males and females respectively in 2002–3, the first year of our analysis. Our standardisation was only for those aged ≥45 years as heart failure before the age of 45 is likely to be due to different aetiology, e.g. congenital heart disease; Teng et al used the population aged ≥20 years for the denominator and a look back period of 10 years to ensure first admission for heart failure, the limitations of our dataset meant we could only apply a two year look back period.

Najafi and colleagues [5] used Australian national data for 2003–4 on all episodes of care for heart failure (both index admissions and readmissions) and age adjustment based on a ‘European’ population standard to derive age-standardised separation rates for heart failure as principal diagnosis of 210 per 100,000 for males and 150 per 100,000 person-years for females. Using similar US hospital discharge data standardised to the 2000 US population, Fang et al [2] reported age-adjusted hospitalisation rates of 390 per 100,000, more than double the Australian estimate. These discrepancies likely reflect the methodological differences in coding procedures, admission policies, and differences in treatment thresholds and practices.

### **Co-morbidities**

The median number of co-morbidities recorded for our cohort was low, regardless of whether the Charlson Index was based on records for the index admission only, or including admissions in the previous two years [17]. Despite likely under-reporting of co-morbidities in the APDC [18], our data showed similar co-morbidity profiles to those reported elsewhere [2,19]. The three most commonly recorded co-morbidities in our data set were diabetes (27%), pulmonary disease (15.4%) and renal failure (14.2%). These co-morbidities are predictors of higher patient treatment costs, particularly in the last six months of life [20,21]. In our study, increasing age, increasing Charlson comorbidity score and male gender were risk factors for hospital readmission.

### **Hospital readmission and deaths**

Overall readmission rates were high. We found readmission rates of 27% at 28 days for any cause (73% at one year) and 11% for admissions due to heart failure (32% at one year) with strong age-related trends. Our estimate of 32% heart failure readmissions at one year is likely to be an underestimate as we only counted readmissions where heart failure was the principal separation code.

Overall mortality rates were 10% at 28 days and 28% at one year, similar to those reported by Teng et al (30-day and one year mortality rates 9.5% and 26.7% respectively) in Western Australia [7] and by Bueno et al in a US Medicare population (30-day mortality rate 10.7% in 2006) [22]. Mortality rates increased consistently with age, and the poor heart failure prognosis was most marked in the older age groups.

### **Length of stay (LOS)**

The mean LOS for the index admission for the entire cohort was 7.8 days, and increased with increasing age, both for the index admission and for readmissions (all cause and for heart failure). Our estimates are higher than some US estimates (mean LOS 4–5 days [1], and 6.4 days in a Medicare population [22]), although comparable to LOS reported in the UK (median LOS 7 days for those aged <75 years and 8 days for those aged ≥75 years [23]). The mean duration of readmissions for heart failure was longer than for any cause and, for most age-groups comparable to the duration of the index admission. This highlights the need to stabilise patients with heart failure before discharge, particularly the oldest patients.

### **Bed-day usage**

Use of acute hospital resources was substantial, with the patients occupying almost 200,000 bed-days per year in NSW over the five year study period. Of these, around 77,000 bed-days per year were attributed to admissions (index or readmissions) where heart failure was the principal separation diagnosis. Had we included readmissions where heart failure was an additional diagnosis and contributory factor, our estimates would have been even higher. Most notable is the impact of the disease on the elderly. The bed-days analysis included 8,370 patients aged ≥85 years. In the first year (index and readmissions), these patients accounted for 106,679 acute hospital bed-days and around 60% of these were for elderly women (66,920/106,679), reflecting the higher proportion of women in the older age groups and the longer LOS for these patients.

### **Typical patient and typical trajectory**

This unique dataset allows us to formulate a “typical” heart failure patient and “typical” trajectory from first hospital admission; these also suggest policy directions for health decision makers.

***The typical patient is aged over 75 years, more likely to be female at this age, and with two co-morbidities, although the incidence has been decreasing over the last 5 years.*** Given that the burden of disease is in the elderly, prevention is the key to controlling this condition. The reduction in CHF may be partly due to the fall in AMI [14], better control of hypertension [24], and adherence to evidence-based guidelines for the management of chronic heart failure [25].

***There is a high all cause re-admission rate, with 27% of patients being readmitted within the first 28 days, and 73% readmitted in the first year; on average 40% of these readmissions are due to CHF, and those readmissions are longer (8.3 days) than the initial admission (7.8 days).*** These figures suggest that chronic disease management plans are important to educate patients about controlling CHF. Community based outreach programs may be nurse-led interventions or involve multidisciplinary teams, with evidence that these heart failure specific management programs can reduce mortality and improve quality of life [26]. However, these multifaceted interventions may not always reduce readmission rates [27], and a large US trial of telemonitoring of heart failure patients failed to show differences in readmissions for any reason or death from any cause with this enhanced patient surveillance [28].

***Patients with CHF use 200,000 bed-days per year in a state with a population of around 6 million people; over 80% of these bed days in the first year after the index admission.*** Failure to adequately stabilise patients before hospital discharge risks early re-admission and, particularly in the elderly, the duration of re-admissions can be long. With hospitals under cost and bed-occupancy pressures, these impacts can be substantial. Intensive specialist clinics and outpatient programs within the first few weeks of first hospital admission may be able to reduce this burden on the hospital system.

***Median survival from time of initial diagnosis for CHF is 1.5 years for those aged ≫85 and about 3 years for those aged 80–85.*** This prognosis is similar to many cancers [29], and highlights the need to address palliative care issues in the management of CHF. Recent studies indicate that medical admissions tend to be concentrated in the last 6 months of life, at least in US settings [20,21], and better palliative care programs and more hospice places may avert these acute admissions.

### **Limitations and strengths of the study**

This study shares the limitations of other data linkage studies, with reliability of study conclusions dependent on the accuracy of the record linkage, the study definitions used and the validity of the coding in the hospital records. A technical assessment of the record linkage quality for this project determined a false positive rate (invalid links) of 0.3% and a false negative rate (missed links) of <0.1%. The administrative data sets used in this study contained no information on the medical management of heart failure, in particular use of drugs such as ACE inhibitors, angiotensin receptor blockers and beta-blockers that have been shown to improve long-term survival.

Examining heart failure presents a particular challenge as it is not a clearly defined disease entity but rather a complex clinical syndrome often following a history of cardiovascular disease or as a complication of diabetes, resulting in ambiguity in its diagnosis and reporting in medical records and hospital separation data. We chose to present readmissions for any cause and those due to heart failure because of the greater reliability of the coding for disease in the primary diagnosis position [30]. We did not refer to the original medical records to confirm a diagnosis of heart failure in the study subjects. However a recent validation study conducted in Western Australia using similar datasets concluded a positive predictive value of 99.5% with a coding of heart failure in the principal diagnostic position [6].

The major strength of this study is that patient-level analyses allowed us to calculate readmission rates, median survival and importantly, quantify the strong age-related trends in incidence of disease, LOS and mortality. Record linkage studies based on the data sets of the CHeReL have no selection biases; analyses are based on records of hospital separations for all residents of NSW in both public and private hospitals.

## **Conclusions**

We found that rates of index admissions for heart failure decreased significantly in both males and females over the study period. Rates of hospital readmission were high and were related to age and the presence of co-morbidities. LOS was longest in the oldest patients and duration of stay similar for both index admissions and readmissions for heart failure. Use of acute hospital resources was substantial, with heart failure patients occupying almost 200,000 bed-days per year in NSW over the five year study period. The strong age-related trends observed have implications for policy planners and decision makers and highlight the importance of stabilising elderly patients before discharge and community-based outreach programs to better manage heart failure and reduce readmissions.

## **Abbreviations**

APDC = Admitted Patient Data Collection; ARIA = Accessibility/Remoteness Index of Australia; CHeReL = Centre for Health Record Linkage; ICD = International Classification of Diseases; LOS = Length of stay; NHMD = National Hospital Morbidity Data; NSW = New South Wales; RBDM = Registry of Births Deaths and Marriages; SEIFA = Socio-Economic Indexes for Areas.

## **Competing interest**

The authors declare that they have no competing interests.

## **Author contributions**

JR, S-AP, JA conceived the study; all authors participated in the study design and interpretation of results; PMcE was responsible for the statistical analyses; JR, DH, JA drafted the manuscript; S-AP, PMcE, KI provided critical review of the manuscript. All the authors read and approved the final manuscript.

## Pre-publication history

The pre-publication history for this paper can be accessed here:

http://www.biomedcentral.com/1472-6963/12/103/prepub
