# Correction: The health services burden of heart failure: an analysis using linked population health data-sets

**DOI:** 10.1186/1472-6963-13-179

**Published:** 2013-05-17

**Authors:** Jane Robertson, Patrick McElduff, Sallie-Anne Pearson, David A Henry, Kerry J Inder, John R Attia

**Affiliations:** 1 School of Medicine and Public Health, The University of Newcastle, Newcastle, Australia; 2 Hunter Medical Research Institute, The University of Newcastle, Newcastle, Australia; 3 UNSW Cancer Research Centre, University of New South Wales and Prince of Wales Clinical School, Sydney, Australia; 4 Institute for Clinical Evaluative Sciences and Department of Medicine, University of Toronto, Toronto, Canada; 5 Clinical Pharmacology, Calvary Mater Hospital, The University of Newcastle, Clinical Sciences Building, Waratah, NSW 2298, Australia

## Correction

After publication of this work [1], we noted that we inadvertently included the wrong version of Table two. The Charlson scores presented in the table of the published paper did not exclude heart failure (as described in the methods). Therefore all estimates of comorbidity burden are inflated by one point. While this changes the absolute values of the comorbidity burden it does not alter the conclusions of the study or the patterns of comorbidity described.

The correct data are shown in the following revised Table two (Table 1 here):

**Table 1:** Co-morbidity burden assessed by Charlson Index

**Variable**	**Statistic**	2002 – 03*	**2003 - 04**	**2004 - 05**	**2005 - 06**	**2006 - 07**
		**(N = 5854)**	**(N = 5935)**	**(N = 5606)**	**(N = 5813)**	**(N = 5953)**
Charlson Score	mean (sd)	1.2 (1.5)	1.2 (1.5)	1.5 (1.6)	1.3 (1.5)	1.4 (1.6)
(based on index admission)	median	1.0	1.0	1.0	1.0	1.0
(q1, q3)	(q1, q3)	(0.0, 2.0)	(0.0, 2.0)	(0.0, 2.0)	(0.0, 2.0)	(0.0, 2.0)
Charlson Score	mean (sd)	1.7 (1.8)	1.8 (1.9)	2.0 (2.0)	1.8 (1.9)	1.9 (2.0)
(based on two years history)	median	1.0	1.0	2.0	2.0	2.0
(q1, q3)	(q1, q3)	(0.0, 3.0)	(0.0, 3.0)	(0.0, 3.0)	(0.0, 3.0)	(0.0, 3.0)

* Financial Year (1 July – 30 June).

† N = number of persons with index admissions.

sd = standard deviation; q1,q3 = quartile 1, quartile 3.

The revised text in the results should read:

### Comorbidity burden

Patients had a median of 1.0 comorbidity recorded at baseline admission, although the range was wide (0–12, not including heart failure), with some evidence of an increase in comorbidity burden over time Table two (Table 1 here). Re-calculation of the Charlson Index from hospital separation codes at the index admission and all admissions in the previous two years combined did not change the estimates substantially. Across the cohort this had the effect of increasing the mean number of comorbidities per patient by 0.5, with the median number of recorded comorbidities increasing from 1.0 to 2.0.

## Pre-publication history

The pre-publication history for this paper can be accessed here:

http://www.biomedcentral.com/1472-6963/13/179/prepub
